# Tuberculosis in Giraffe

**Published:** 1892-03

**Authors:** J. C. Meyers


### TUBERCULOSIS IN GIRAFFE.

By J. C. Meyers, Sr., V. S.

Four weeks ago when Dr. Meyers, Jr., was called to treat the male giraffe at the Zoological Gardens, Cincinnati, the symptoms indicated a catarrhal affection of the lungs; upon the third visit, however, he informed Supt. Mr. Sol. Stevens, that he suspected it to be tuberculosis, whereupon Pres. Mr. A. E. Burkhardt became alarmed, and concluded to ask for a consultation, and telegraphing to the editors of the Journal asking whom they should employ, received the answer, "Meyers Senior and Junior, Cincinnati," so that I was called in.

The symptoms of the patient were actually such that I was obliged to coinside with the given diagnosis.

Prophylaxis, to protect, if possible, the female that is housed in the same enclosure, and that seems to be in good health, was immediately ordered.

In order to be positive in regard to diagnosis. Some of the sputum which "Abe" expectorated was taken, and given to a bacteriologist for examination, who discovered a great number of bacilli. Hope of recovery being impossible, an unfavorable prognosis was given.

Death ensued on the night between the 20th-21st inst, thirteen days after my first visit, and about four months after taking sick.

Post mortem examination, which was made about 12 hours after death, substantiated the correctness of the diagnosis.

Each lobe of the lungs presented a conglomeration of an almost solid mass, to which the tuberculous tumors, particularly those filled with a yellowish, cheesy corruption, added the most material, increasing the circumference of the lungs greatly. Upon cutting through some portion of the lungs, a gritty sound was audible, presumably due to a deposition of lime. The liver in its parenchyma contained many small-sized tubercles. The spleen and other abdominal organs were exempt.

A renewed search in another quantity of sputum, as also in the decomposed tubercles, revealed numerous bacilli, particularly in the mucus.

